# Megafauna Seed Dispersal in the Neotropics: A Meta-Analysis Shows No Genetic Signal of Loss of Long-Distance Seed Dispersal

**DOI:** 10.3389/fgene.2019.00788

**Published:** 2019-09-05

**Authors:** Rosane G. Collevatti, Jacqueline S. Lima, Liliana Ballesteros-Mejia

**Affiliations:** Laboratório de Genética and Biodiversidade, ICB, Universidade Federal de Goiás (UFG), Goiânia, Brazil

**Keywords:** angiosperms, genetic diversity, general linear mixed models, meta-analysis, Neotropics, phylogenetic generalized least square models, phylogenetic meta-analysis, phylogenetic signal

## Abstract

Restricted gene flow may lead to the loss of genetic diversity and higher genetic differentiation among populations, but the genetic consequences of megafauna extinction for plant populations still remain to be assessed. We performed a phylogenetic-independent meta-analysis across 102 Neotropical plants to test the hypothesis that plant species with megafaunal seed dispersal syndrome have a lower genetic diversity and a higher genetic differentiation than those without it. We classified as megafauna-dependent plant species those that potentially relied only on megafauna to seed dispersal, and as megafauna-independent those that relied on megafauna and other seed dispersers. Our data comprised 98 studies using microsatellite markers. We found no statistical difference in genetic diversity and differentiation between plants with megafauna and non-megafauna seed dispersal syndrome, although the statistical power to detect differences in genetic differentiation was low. Moreover, we found no statistical difference between megafauna-dependent and megafauna-independent plant species. We then used generalized linear mixed models and phylogenetic generalized least square models to investigate the effects of megafaunal seed dispersal syndromes and reproductive traits on variation in genetic diversity and genetic differentiation. We found no effect of megafaunal syndrome, rather, reproductive traits, such as pollination mode, mating, and breeding systems, showed significant effects. Our findings show that the genetic studies of Neotropical plants performed so far show no difference in genetic diversity and differentiation in plants with megafaunal compared to those with non-megafaunal seed dispersal syndromes. Our results also provide evidence pointing out that plant species with megafaunal seed dispersal syndromes may have used different strategies to counterbalance the extinction of their mutualistic megafauna dispersers, such as the dispersal by extant mammals that may promote long-distance seed dispersal. Our results also reinforce the importance of pollination to long-distance gene flow in Neotropical plants.

## Introduction

Neotropical plants producing large fruits and seeds or seeds with thick and hard endocarps or coats embedded in indehiscent fruits are known as biological anachronisms (Janzen and Martin, 1982), adapted to a now extinct megafauna (mammals >1,000 kg) that once dominated Pleistocene Neotropical landscapes. Nowadays, seeds of several Neotropical species matching Janzen and Martin’s (1982) megafauna syndrome remain below their mother trees without being dispersed. It raises the hypothesis that these plants have lost their seed dispersers, especially the long-distance seed-dispersing species. Megafauna species may have promoted long-distance dispersal by massive seed dispersal (Pires et al., 2017), that is, defecating large amounts of seeds over large areas, or by carrying large individual seeds over long distances (Janzen and Martin, 1982; Guimarães et al., 2008; Hansen and Galetti, 2009; Pires et al., 2017).

In the Neotropics, fruits of megafauna syndrome species are consumed by many extant mammals, such as agouti, but only extant large mammals, such as tapirs, large bats, Atelinae monkeys, manned wolf, and deer, can act as long-distance seed dispersers by endozoochory (Tabarelli and Peres, 2002; Guimarães et al., 2008; Hansen and Galetti, 2009). In addition, germination rates of seeds swallowed and defecated by larger mammals may be higher than those of seeds consumed by smaller mammals because of greater scarification (Westoby et al., 1996; Moles and Westoby, 2004; Bodmer and Ward, 2006).

Even though extant frugivores may disperse seeds with megafaunal syndrome, they might not counterbalance the effects of the extinct megafauna species following the biological anachronism reasoning (Guimarães et al., 2008; Hansen and Galetti, 2009) because of both smaller body sizes and gut retention time (Van Soest, 1996; Clauss et al., 2007; Clauss et al., 2008). Predictions of the effects of megafauna extinction include a decrease in the number of seeds successfully dispersed away from the maternal plant, a decrease in recruitment because of high mortality of nondispersed seeds close to the mother plant, and restricted gene flow because of the loss of long-distance dispersal agents (Janzen and Martin, 1982; Collevatti et al., 2003; Guimarães et al., 2008; Hansen and Galetti, 2009). Nevertheless, plant species linked to megafauna syndromes may have counterbalanced the extinction of their long-distance seed dispersers by relying on scatter-hoarding rodents (Jansen et al., 2012).

Megafauna herbivores may have also affected community structure because of their foraging behavior, causing disturbance in vegetation community, creating gaps, and reducing vegetation density, thus increasing spatial heterogeneity (Johnson, 2009). Because of high deposition of urine and feces, they might also have increased nutrient recycling, affecting community structure, and reduced fire frequency because of grazing (e.g., Hester et al., 2006; Gill et al., 2009; Gill, 2014; Malhi et al., 2016). Although some of the consequences of the extinction of seed dispersers have been assessed for community structure (Hester et al., 2006; Gill et al., 2009), nutrient cycling (Malhi et al., 2016) and plant-frugivore interactions (Gill, 2014), the genetic consequences for plant populations still remain to be evaluated. Many authors have predicted that the loss of megafauna dispersal may lead to a lower gene flow among populations, leading in turn to the loss of genetic diversity and higher genetic differentiation among populations (e.g., Collevatti et al., 2003; Guimarães et al., 2008; Pires et al., 2014; Malhi et al., 2016); however, those predictions have not been tested so far. Alternatively, the effects of reproductive traits (RT), such as pollination, mating, or breeding mode, have not been tested either. In the Tropics, plant species with outcrossing mating systems show mixed results regarding genetic differentiation (Bawa, 1992). Pollination modes and mating systems have been related to patterns of genetic diversity and differentiation in Neotropical plants (Ballesteros-Mejia et al., 2016). For instance, Neotropical plants with outcrossing mating systems have a lower genetic differentiation (*F* *_ST_*), and beetle-pollinated species have a higher allelic richness (*AR*) (Ballesteros-Mejia et al., 2016). Thus, RT may account for differences in genetic diversity and differentiation among Neotropical plants, despite seed dispersal syndrome (Ballesteros-Mejia et al., 2016).

Herein, we address whether Neotropical plant species linked to megafauna syndromes have indeed a lower genetic diversity and a higher genetic differentiation among populations using a phylogenetic meta-analysis framework. In addition, we test whether megafauna seed dispersal syndrome or other RT, such as breeding system, mating system, and pollination mode, are responsible for variations in genetic diversity and differentiation among species. We model the variation in genetic parameters using generalized linear mixed models (GLMM) and phylogenetic generalized least squares (pGLS) to account for phylogenetic non-independence. Our analyses are based on published data on the polymorphism at nuclear microsatellite molecular markers.

## Materials and Methods

### Data Selection

We compiled our database by performing an exhaustive search for published studies on population genetics of Neotropical plant species. We used the online databases Web of Science platform (ISI, www.webofknowledge.com), Scopus (http://www.elsevier.com/online-tools/scopus), and Portal de Periódicos Capes (http://www.periodicos.capes.gov.br). The online search engine Google (www.scholar.google.com) was also used to identify scientific reports, theses, and other gray literature. Our data compilation includes publications dating from 1945 (first register in ISI) to December 2016. We carried out the search with the most common keywords in the area: “phylogeography,” “population genetics,” “genetic diversity,” and “genetic structure,” combined with (and) “Neotropics,” “Neotropical tree*,” “Neotropical plant*.” We considered the Neotropics as the region comprising the Neotropical floristic region (Cox and Moore, 1985), including southern Florida, lowlands in Mexico, Central America, Caribe, and South America. We retained only the studies that estimated genetic parameters using nuclear microsatellite markers to avoid the effect of different evolution modes among molecular markers.

We first classified plant species into two categories: species that exhibit megafauna dispersal syndrome, that is, those species that had their seeds potentially dispersed by megafauna, and species that do not exhibit megafauna syndrome, that is, those species that have their seeds dispersed by other factors (wind, water, authochory) or animal species (zoochory) but not megafauna (**Table S1**). Moreover, we classified plant species as megafauna dependent, that is, species that potentially relied only on megafauna to disperse, and megafauna independent, that is, species that relied on megafauna and other seed dispersers (**Table S1**). To classify the plant species, we used the definitions proposed by Guimarães et al. (2008) and an expert opinion (Mauro Galetti, personal communication). Thus, we followed the literature on megafauna syndrome classification, avoiding new classifications that could lead to data misinterpretation. Guimarães et al. (2008) defined megafauna syndrome fruits as small or large fleshy fruits (4–10 cm in diameter) with up to five large seeds (generally >2.0 cm diameter), which they call “type I fruits,” and large fleshy fruits (> 10 cm diameter) with many small seeds (> 100), which they call “type II fruits.” This classification excludes large fruits without a fleshy pulp. In our data set, megafauna-dependent species includes “type I fruits” (**Table S1**).

To model the effects of megafauna seed dispersal syndromes and other RT on variations in genetic diversity and genetic differentiation among populations, we compiled information about RT, such as mating and breeding systems and pollination mode. Trait data were obtained from botanical reviews and articles about pollination system and plant reproduction. We then classified the species according to their pollination syndrome as anemophily (pollinated by wind), chiropterophily (pollinated by bats), entomophily (pollinated by insects), and ornithophily (pollinated by birds). For mating systems, we found species with outcrossing and mixed systems. For breeding systems, we found monoecious, dioecious, and hermaphrodite species.

### Genetic Parameters

To measure genetic differentiations, we compiled Wright’s (1951) FST. To measure genetic diversity, we obtained Nei’s (1978) genetic diversity (He) and AR (Mousadik and Petit, 1996). As articles often do not report the same genetic parameters, sample sizes might vary among the parameters analyzed.

### Effect of Megafauna Seed Dispersal Syndromes in Genetic Parameters

To analyze the effect of megafauna seed dispersal syndromes on genetic diversity and differentiation of Neotropical plant species, we divided the whole database into three different sets to account for differences in seed size and morphology caused by the dispersal syndrome. For example, hydrochoric species usually have large seeds, whereas autochoric species usually have very small seeds compared to mammal-dispersed seeds. Set 1 included only species dispersed by mammals, set 2 included only species with zoochoric dispersal syndrome (including mammals), and set 3 included all species in the data sets, that is, zoochoric, wind-dispersed, autochoric, and hydrochoric species.

Reproductive and seed traits related to seed dispersal mode (e.g., seed size and number) in Angiosperm have significant phylogenetic signal and evolutionary constraints imposed by reproductive structures (Jordano, 1995). Many traits in seed plants have evolutionary interdependencies that may constrain evolutionary and adaptive responses (Donoghue, 1989). Thus, for each of the three data sets, we performed phylogenetic non-independent (PH-NoIND) and phylogenetic-independent (PH-IND) meta-analyses (Lajeunesse, 2009) using random-effect models, fitting species identity as a random factor. We compared the overall pooled effect sizes (δ) for each data set using Hedges’s *d* (Hedges and Olkin, 1985), which measures the magnitude and direction of experimental outcomes in standardized units. We obtained their variances and 95% confidence intervals (CI) and performed the Z-test for non-zero effects (Hedges, 1992; Hedges and Olkin, 1985). The Z value is the weighted sum of square caused by the regression model (Hedges, 1992). Random-effect models assume that replicates come from different distributions and add an additional variance component (τ) to each replicate as an estimate of between-replicate variances. We evaluated the fit of PH-NoIND and PH-IND meta-analysis models using Akaike information criterion (AIC) based on Butler and King (2004). The model with the lowest AICc (AIC corrected by sample size and number of parameters; Burnham and Anderson, 2002) was considered as the more plausible to explain observed patterns. Analyses were performed using *phyloMeta* v.1.2 (Lajeunesse, 2011) implemented in R v. 3.3.2 (R Core Team, 2015).

### Phylogenetic Analysis

To account for phylogenetic non-independence of megafauna syndromes and RT on meta-analysis and on regression-based models (see below), we built a phylogenetic hypothesis of all species included in the analysis using the internal master tree *Phylomatic treeR20120829* from the platform *Phylomatic* (Webb and Donoghue, 2005). In the absence of information about branch length, it was set to a value of 1. Most of the phylogenetic relationships within plants at deeper levels are well–resolved; therefore, polytomies at terminal nodes (species level) would not affect the results (Purvis and Garland, 1993; Swenson, 2009).

### Syndromes in Genetic Parameters

Because PH-NoIND meta-analysis models fitted better for all three data sets (see *Results*), yielding very similar results to phylogenetic independent meta-analyses, we only mentioned important data for PH-IND in the main text but presented details in the Supporting Information (**Figures S1** and **S2**, **Tables S3**–**S5**). We tested whether species with megafauna seed dispersal syndromes have a higher genetic differentiation and a lower genetic diversity compared to species without megafauna syndrome with PH-NoIND meta-analysis. For each data set and genetic parameter, we assessed the mean difference and 95% CI (Schwarzer et al., 2015) between species with megafauna seed dispersal syndromes and no megafauna syndromes. We also compared megafauna-dependent versus megafauna-independent plant species and megafauna-dependent versus species with no megafauna syndromes. Analyses were performed using *meta* package (Schwarzer et al., 2007; Schwarzer et al., 2015) implemented in R v. 3.3.2 (R Core Team, 2015). Moreover, we ran a power analysis to determine the statistical power of our comparison given the restrictions in sample size (Valentine et al., 2010; Quintana, 2015). This analysis was performed with *metapower* R-script (Quintana and Tiebel, 2018).

### Modeling the Effect of Megafauna Seed Dispersal Syndromes and Reproductive Traits on Genetic Parameters

To investigate the effects of megafauna seed dispersal syndromes and RT on genetic diversity and differentiation, we used generalized linear mixed models (GLMM). Pollination mode and breeding system were treated as multistate categorical variables, whereas mating system (i.e., mixed or outcrossing) and megafauna syndromes (yes or no) were treated as binary variables. RT and megafauna syndromes were fitted as *fixed factors*, whereas species identity was fitted as *random factors*. When a species was studied with the same marker more than once, we calculated the average values for each genetic parameter (10 cases in 102 species). We fitted separate models for each genetic parameter (*F* *_ST_*, *He*, and *AR*) and for each one of the three data sets. Analyses were carried out using *MCMCglmm package* (Hadfield, 2010) implemented in R version 3.3.2 (R Core Team, 2015) that uses a Bayesian framework with Markov Chain and Monte Carlo approximation algorithms. It was run with a Gausian distribution and a total of 80,000 iterations (burn-in 20,000 chains). Results were summarized by the mean of the posterior distribution and 95% CI, indicating direction, strength, and significance of the effects. Modeling of megafauna-dependent and -independent species was not possible because of the small sample size.

We used the phylogenetic tree to test whether megafauna seed dispersal syndromes and RT have a phylogenetic signal for the species in our data sets, that is, phylogenetic-related species tend to be more similar than expected by chance (Blomberg and Garland, 2002) using the Abouheif’s (Abouheif, 1999; Pavoinea et al., 2008) proximity test implemented in the R *package adephylo* (Jombart et al., 2010). Then, we fitted pGLS models (Martins and Hansen, 1997) to verify whether results obtained by the GLMM were robust enough so the pattern persisted after accounting for phylogenetic relationships. pGLS analyses were carried out using the *package carper* (Orme et al., 2012) in R environment.

## Results

### Effect of Megafauna Seed Dispersal Syndromes in Genetic Parameters

We used a database with a total of 102 species (**Table S1**) to perform PH-NoIND and PH-IND meta-analyses, comprising 98 studies (**Table S2**). All effect sizes were significantly different from zero for both metrics of meta-analyses and for all data sets (p < 0.001 for all comparisons, **Figures S1** and **S2**, **Tables S3**–**S5**). PH-NoIND meta-analyses’ metrics showed lower AICc (Akaike information criterion corrected for sample size and number of parameters) and fitted better to our data for all analyses (**Figures S1** and **S2**, **Tables S3–S5**).

**Figure 1:**
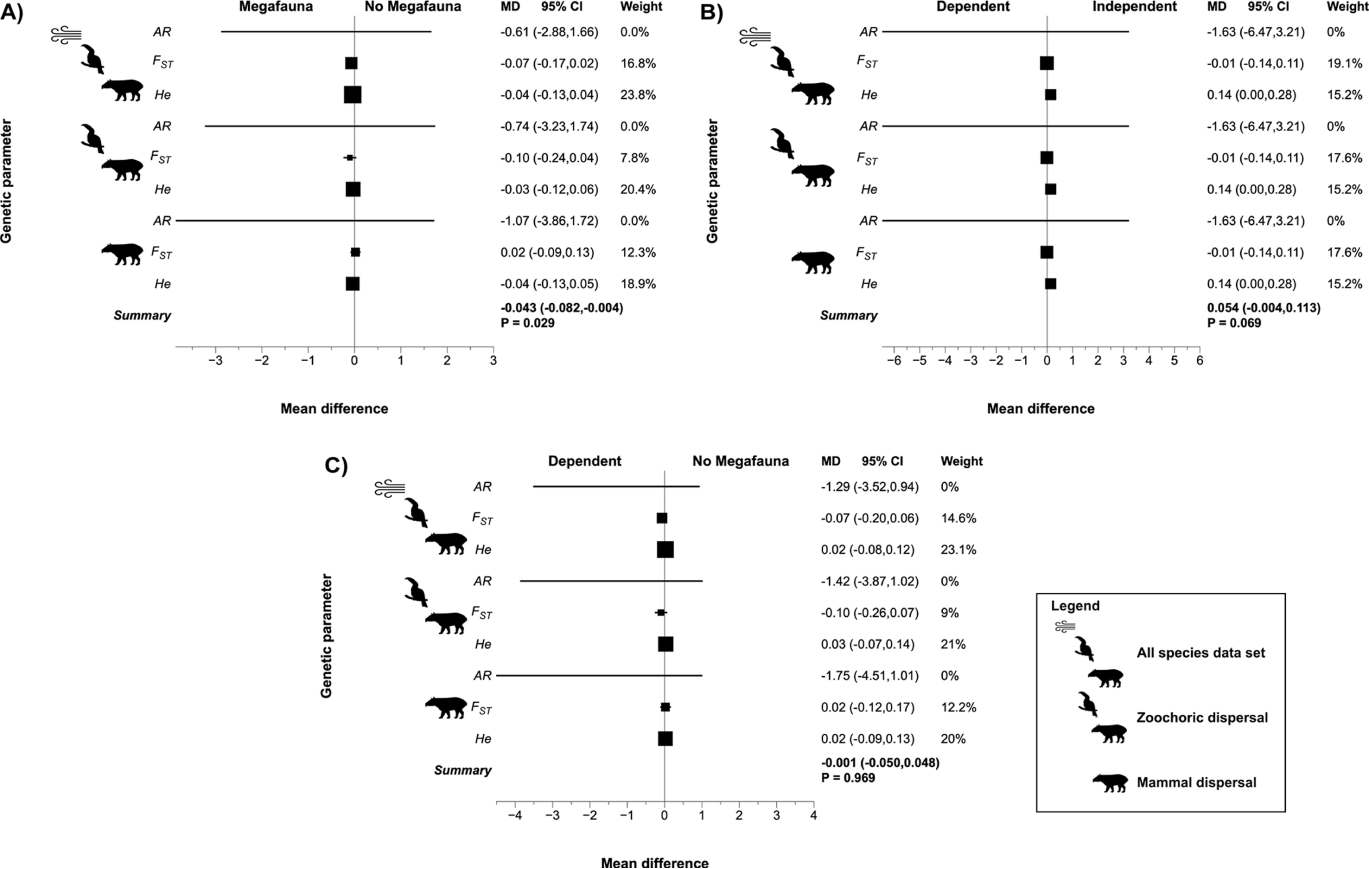
Effect of megafauna seed dispersal syndromes on genetic parameters across the three data sets for plant species with megafauna and no megafauna syndromes based on phylogenetic-dependent meta-analysis. **(A)** Effect size for species with megafauna versus no megafauna syndrome dispersal. **(B)** Effect size for megafauna-dependent versus megafauna-independent species. **(C)** Effect size for megafauna-dependent versus no megafauna syndrome species.

Contrary to expectation, metrics of PH-NoIND meta-analyses showed no significantly higher genetic differentiation for species with megafauna seed dispersal syndrome than for species without megafauna syndrome (**Figure 1A**, **Table S6**) for all data sets. However, power analyses reveal that statistical power given both effect size and sample size is low (**Tables S3**–**S5**). Furthermore, none of the genetic diversity parameters studied (*He* and *AR*) was significantly lower for species with megafauna syndrome than for no megafauna syndrome (**Figure 1A**, **Table S6**). Megafauna-dependent and megafauna-independent plant species showed no significant differences in any of the genetic parameters studied (**Figure 1B**, **Table S6**). Moreover, megafauna-dependent and plants with no megafauna syndrome also showed no significant differences in any of the genetic parameters analyzed (**Figure 1C**, **Table S6**). Power analysis indicates that results for genetic diversity parameters (*He* and *AR*) have enough statistical power (**Tables S3**–**S5**) to support results of the meta-analysis.

### Modeling the Effect of Megafauna Seed Dispersal Syndromes and Reproductive Traits on Genetic Parameters

RT rather than megafauna seed dispersal syndromes showed significant effects on genetic parameters for Neotropical plant species in GLMMs.

Genetic differentiation (*F* *_ST_*) was lower for plant species with outcrossing mating system when all data sets or only zoochoric species were analyzed (**Tables S7**–**S9**). Genetic diversity (*He*) was significantly lower for species pollinated by insects when considering only mammal-dispersed or zoochoric species (**Tables S7**–**S9**) and significantly higher for species with outcrossing mating systems. *AR* was not significantly related to any RT studied.

Megafauna seed dispersal syndromes and pollination mode showed significant phylogenetic signals in our data sets (**Table S10**). However, mating and breeding systems had no phylogenetic signal most likely caused by incomplete taxon sampling because few species have been studied. Analyses accounting for phylogenetic non-independence (pGLS) confirmed the results of GLMM for genetic differentiation (**Tables S11**–**S12**). *F* *_ST_* values are lower for species with outcrossing mating systems, considering all data sets and zoochoric species data set (**Tables S11**–**S12**). Genetic diversity (*He*) was significantly higher for species with outcrossing system for all data sets (**Tables S11**–**S12**), but the relationship of *He* and pollination mode was not recovered.

## Discussion

Our findings show no significant signal of megafauna extinction in genetic diversity and differentiation of Neotropical plants. Plants with megafaunal seed dispersal syndromes showed similar values of genetic differentiation and genetic diversity compared to species without megafaunal dispersal syndromes. Moreover, the lack of genetic signal was not caused by the dependency level on megafauna dispersal as megafauna-dependent species showed no difference in genetic parameters when compared to species with non-megafaunal syndromes or to megafauna-independent species. It is important to note that statistical power was low for genetic differentiation comparisons but very high for the other genetic parameters.

The lack of genetic signal may be caused by the dispersal rescue effect of other disperser species. Extant mammals, such as tapir and deer, may move seeds over long distances and may have long enough gut retention, potentially leading to long-distance dispersal (e.g., Vellend et al., 2003; Clauss et al., 2010). In addition, the role of scatter-hoarding rodents in long-distance dispersal has been underestimated until recently (Jansen et al., 2012), and these mammals may be important in long-distance dispersal to many Neotropical plant species, counterbalancing the past role of the extinct megafauna, maintaining genetic diversity and differentiation among populations at the same levels of species with non-megafaunal syndromes.

Furthermore, pollination may be more important than seed dispersal in shaping patterns in genetic differentiation and diversity in the Neotropics. In fact, many works show higher contribution of pollen dispersal than seed dispersal to gene flow in Angiosperms (e.g., Petit et al., 2005) and in Neotropical plants (e.g., Hamilton and Miller, 2002; Collevatti et al., 2003; Ballesteros-Mejia et al., 2016). Indeed, our findings show significant effects of RT, such as pollination mode, mating, and breeding systems, in genetic differentiation and diversity, strengthening the importance of RT in long-distance dispersal (Ballesteros-Mejia et al., 2016).

It is important to note that the lack of evidence of an effect of megafauna seed dispersal syndrome on genetic diversity and differentiation does not represent evidence of a lack of effect of megafauna because confounding effects, such as species demographic history, may hinder the detection of any genetic signal of loss of long-distance seed dispersal. The extinction of megafauna may be relatively recent compared to the generation time of the plant species and may not be long enough to generate a genetic effect. Most species studied so far are trees (89 in 102 species) with 15–20 years of generation time (i.e., time to first reproduction). The last megafauna extinct at *c*. 6 ka, corresponding to *c*. 400 generations, which may not be enough to reduce genetic diversity and increase genetic differentiation in long-lived trees. In addition, many Neotropical trees experienced retraction or expansion in both their geographical range and effective population size during glacial periods, such as the species with megafaunal seed dispersal syndromes *Caryocarbrasiliense* (Collevatti et al., 2012), *Dipteryxalata* (Collevatti et al., 2013), and *Mauritia flexuosa* (Lima et al., 2014) and the species with non-megafaunal syndromes *Tabebuia impetiginosa* (Collevatti et al., 2012), *Dalbergiamiscolobium* (Novaes et al., 2013), *Tabebuia aurea* (Collevatti et al., 2015), and *Tabebuia roseoalba* (Melo et al., 2016). Thus, demographic history may have also shaped the current geographical distribution of genetic diversity and differentiation, bewildering the effects of megafauna extinction. The data available so far have not enough statistical power to detect effects of megafauna syndrome in genetic differentiation. Hence, future studies may shed more light on the effects of megafauna extinction in genetic differentiation.

It is worth noticing however that the definition of megafauna seed dispersal syndromes is controversial because it includes fruits and seeds with highly contrasting characteristics (Howe, 1985). For instance, plants with fruits and large seeds, such as *Mauritia flexuosa*, *Dipteryxalata*, *Caryocarbrasiliense*, are considered species with megafauna seed dispersal syndromes but so are plants with large or medium fruits with small hard-coated seeds, such as *Annona crassiflora*, *Annanascomosus*, *Enterolobiumcyclocarpum*, and *Solanum lycocarpum* (Janzen and Martin, 1982; Guimarães et al., 2008). These megafauna syndrome species with very different fruit and seed characteristics may have used different dispersal agents for long-distance dispersal to counterbalance the extinction of their megafauna mutualistic seed dispersers or may rely on secondary dispersers that improve long-distance dispersal (Levey et al., 2002). In addition, megafauna seed dispersal syndromes may be adaptations to another environmental condition. For instance, large seed size may also be the outcome of adaptation to drought or nutrient-poor soils (Westoby et al., 1996; Moles and Westoby, 2004), which may explain large seeds in many savanna species. Species with megafauna syndromes may also have survived the extinction of megafauna *via* other traits that also promote long-distance dispersal and recruitment but are not related to fruit or seed characteristics (Johnson, 2009). For example, many Neotropical species have high capacity for vegetative propagation or sprouting or are long lived, such as most savanna tree species, or are able to establish beneath the parent plant (Donatti et al., 2007; Collevatti and Hay, 2011).

In conclusion, the genetic studies of Neotropical plants show no difference in genetic diversity and differentiation in species with megafaunal seed dispersal syndromes compared to non-megafaunal. Rather, a combination between RT, such as pollination mode and mating and breeding systems, along with demographic history may be more important in shaping the current genetic diversity and differentiation patterns in Neotropical plants.

## Author Contributions

RC conceived and funded the work. RC and JL obtained the data. LB-M and JL carried out analyses. RC wrote the original draft, and all authors contributed to the manuscript and approved the final version.

## Funding

This work was supported by the research network Rede Cerrado CNPq/PPBio (project no. 457406/2012-7), PROCAD/CAPES (project no 88881.068425/2014-01) and CAPES Ciências sem Fronteira (project CSF-PAJT/CAPES no. 88881.030318/2013-01). JSL receives a fellowship from PROCAD/CAPES and LBM receives a fellowship from CAPES Ciências sem Fronteira.

## Conflict of Interest Statement

The authors declare that the research was conducted in the absence of any commercial or financial relationships that could be construed as a potential conflict of interest.

## Supplementary Material

Supporting information

Supporting Information S1 Tables S1–S12 with information of data set and meta-analysis and GLMM and pGLS results.

Supporting Information S2 Figures S1 and S2 with information of meta-analysis results.
