# Supplementary material for: Megafauna Seed Dispersal in the Neotropics: A Meta-Analysis Shows No Genetic Signal of Loss of Long-Distance Seed Dispersal
Source: Front Genet. 2019 Sep 5;10:788. doi: 10.3389/fgene.2019.00788 (PMC6739635; doi:10.3389/fgene.2019.00788)
Supplement: Supporting Information S1 — Tables S1–S12 with information of data set and meta-analysis and GLMM and pGLS results. [file Table_1.docx]

**Megafauna seed dispersal in the Neotropics: A meta-analysis shows no genetic signal of loss of long-distance seed dispersal**

Rosane G Collevatti*, Jacqueline S Lima, Liliana Ballesteros-Mejia

**Supporting Information S1 - Tables**

**Table S1. Neotropical plant species (102) for which population genetic data are available and their seed dispersal syndrome classification.** ms, megafauna syndrome. md, megafauna syndrome dependence. ms, megafaunal syndrome (ms); no megafauna syndrome (nm); md, megafauna dependent (md), megafauna independent (mi).

| **Family** | **Genus** | **Species** | **MS** | **MD** |
| --- | --- | --- | --- | --- |
| Acanthaceae | *Avicennia* | *Avicennia bicolor* | nm | nm |
| Acanthaceae | *Avicennia* | *Avicennia germinans* | nm | nm |
| Acanthaceae | *Avicennia* | *Avicennia schaueriana* | nm | nm |
| Anacardiaceae | *Myracrodruon* | *Myracrodruon urundeuva* | nm | nm |
| Anacardiaceae | *Anacardium* | *Anacardium humile* | nm | nm |
| Annonaceae | *Annona* | *Annona cherimola* | ms | mi |
| Annonaceae | *Annona* | *Annona coriacea* | ms | mi |
| Annonaceae | *Annona* | *Annona crassiflora* | ms | mi |
| Apocynaceae | *Himatanthus* | *Himatanthus drasticus* | nm | nm |
| Arecaceae | *Acrocomia* | *Acrocomia aculeata* | ms | md |
| Arecaceae | *Attalea* | *Attalea phalerata* | ms | mi |
| Arecaceae | *Bactris* | *Bactris gasipaes* | ms | md |
| Arecaceae | *Butia* | *Butia eriospatha* | ms | md |
| Arecaceae | *Ceroxylon* | *Ceroxylon echinulatum* | nm | nm |
| Arecaceae | *Chamaedorea* | *Chamaedorea ernesti-augusti* | nm | nm |
| Arecaceae | *Euterpe* | *Euterpe edulis* | nm | nm |
| Arecaceae | *Mauritia* | *Mauritia flexuosa* | ms | md |
| Arecaceae | *Oenocarpus* | *Oenocarpus bataua* | nm | nm |
| Bignoniaceae | *Jacaranda* | *Jacaranda copaia* | nm | nm |
| Bignoniaceae | *Tabebuia* | *Tabebuia aurea* | nm | nm |
| Bignoniaceae | *Handroanthus* | *Handroanthus impetiginosus* | nm | nm |
| Bignoniaceae | *Handroanthus* | *Handroanthus ochraceus* | nm | nm |
| Bignoniaceae | *Handroanthus* | *Handroanthus serratifolius* | nm | nm |
| Bignoniaceae | *Handroanthus* | *Handroanthus chrysothrichus* | nm | nm |
| Bignoniaceae | *Tabebuia* | *Tabebuia roseoalba* | nm | nm |
| Bromeliaceae | *Alcantarea* | *Alcantarea geniculata* | nm | nm |
| Bromeliaceae | *Alcantarea* | *Alcantarea glaziouana* | nm | nm |
| Bromeliaceae | *Alcantarea* | *Alcantarea imperialis* | nm | nm |
| Bromeliaceae | *Alcantarea* | *Alcantarea regina* | nm | nm |
| Bromeliaceae | *Pitcairnia* | *Pitcairnia albiflos* | nm | nm |
| Bromeliaceae | *Pitcairnia* | *Pitcairnia staminea* | nm | nm |
| Bromeliaceae | *Vriesea* | *Vriesea minarum* | nm | nm |
| Bromeliaceae | *Orthophytum* | *Orthophytum ophiuroides* | nm | nm |
| Bromeliaceae | *Encholirium* | *Encholirium horridum* | nm | nm |
| Cactaceae | *Polaskia* | *Polaskia chichipe* | nm | nm |
| Cactaceae | *Stenocereus* | *Stenocereus stellatus* | nm | nm |
| Cactaceae | *Pilosocereus* | *Pilosocereus aurisetus* | nm | nm |
| Cactaceae | *Pilosocereus* | *Pilosocereus machirisii* | nm | nm |
| Cactaceae | *Uebelmannia* | *Uebelmannia pectinifera* | nm | nm |
| Caryocaraceae | *Caryocar* | *Caryocar brasiliense* | ms | md |
| Caryocaraceae | *Caryocar* | *Caryocar microcarpum* | ms | md |
| Caryocaraceae | *Caryocar* | *Caryocar villosum* | ms | md |
| Clusiaceae | *Symphonia* | *Symphonia globulifera* | nm | nm |
| Dilleniaceae | *Curatella* | *Curatella americana* | nm | nm |
| Ebenaceae | *Diospyros* | *Diospyros hispida* | ms | mi |
| Fabaceae | *Copaifera* | *Copaifera langsdorffii* | nm | nm |
| Fabaceae | *Dalbergia* | *Dalbergia nigra* | nm | nm |
| Fabaceae | *Dicorynia* | *Dicorynia guianensis* | nm | nm |
| Fabaceae | *Dinizia* | *Dinizia excelsa* | nm | nm |
| Fabaceae | *Dipteryx* | *Dipteryx alata* | ms | md |
| Fabaceae | *Dipteryx* | *Dipteryx panamensis* | ms | md |
| Fabaceae | *Hymenaea* | *Hymenaea courbaril* | ms | md |
| Fabaceae | *Hymenaea* | *Hymenaea stigonocarpa* | ms | md |
| Fabaceae | *Plathymenia* | *Plathymenia reticulata* | nm | nm |
| Fabaceae | *Platypodium* | *Platypodium elegans* | nm | nm |
| Fabaceae | *Vouacapoua* | *Vouacapoua americana* | nm | nm |
| Fabaceae | *Dimorphandra* | *Dimorphandra wilsonii* | ms | mi |
| Fabaceae | *Dimorphandra* | *Dimorphandra mollis* | ms | mi |
| Fabaceae | *Pouteria* | *Pouteria reticulata* | nm | nm |
| Fabaceae | *Parkia* | *Parkia panurensis* | nm | nm |
| Gomortegaceae | *Gomortega* | *Gomortega keule* | ms | mi |
| Lauraceae | *Sextonia* | *Sextonia rubra* | nm | nm |
| Malvaceae | *Luehea* | *Luehea seemannii* | nm | nm |
| Malvaceae | *Theobroma* | *Theobroma grandiflorum* | ms | md |
| Melastomataceae | *Tibouchina* | *Tibouchina papyrus* | nm | nm |
| Melastomataceae | *Miconia* | *Miconia affinis* | nm | nm |
| Meliaceae | *Carapa* | *Carapa guianensis* | nm | nm |
| Meliaceae | *Carapa* | *Carapa surinamensis* | nm | nm |
| Meliaceae | *Cedrela* | *Cedrela fissilis* | nm | nm |
| Meliaceae | *Cedrela* | *Cedrela odorata* | nm | nm |
| Meliaceae | *Swietenia* | *Swietenia humilis* | nm | nm |
| Meliaceae | *Swietenia* | *Swietenia macrophylla* | nm | nm |
| Moraceae | *Bagassa* | *Bagassa guianensis* | ms | md |
| Moraceae | *Ficus* | *Ficus insipida* | nm | nm |
| Moraceae | *Ficus* | *Ficus citrifolia* | nm | nm |
| Moraceae | *Ficus* | *Ficus obtusifolia* | nm | nm |
| Moraceae | *Ficus* | *Ficus yoponensis* | nm | nm |
| Myristicaceae | *Virola* | *Virola surinamensis* | nm | nm |
| Myristicaceae | *Virola* | *Virola koschnyi* | nm | nm |
| Myrtaceae | *Eugenia* | *Eugenia dysenterica* | ms | md |
| Myrtaceae | *Eugenia* | *Eugenia uniflora* | nm | nm |
| Myrtaceae | *Eugenia* | *Eugenia pyriformis* | nm | nm |
| Myrtaceae | *Eugenia* | *Eugenia brasiliensis* | nm | nm |
| Myrtaceae | *Eugenia* | *Eugenia francavilleana* | nm | nm |
| Orchidaceae | *Epidendrum* | *Epidendrum fulgens* | nm | nm |
| Orchidaceae | *Epidendrum* | *Epidendrum cinnabarium* | nm | nm |
| Orchidaceae | *Epidendrum* | *Epidendrum puniceoluteum* | nm | nm |
| Orchidaceae | *Epidendrum* | *Epidendrum secundum* | nm | nm |
| Orchidaceae | *Cattleya* | *Cattleya liliputana* | nm | nm |
| Podocarpaceae | *Podocarpus* | *Podocarpus sellowii* | nm | nm |
| Polygonaceae | *Triplaris* | *Triplaris cumingiana* | nm | nm |
| Rhizophoraceae | *Rhizophora* | *Rhizophora harrisonii* | nm | nm |
| Rhizophoraceae | *Rhizophora* | *Rhizophora mangle* | nm | nm |
| Rhizophoraceae | *Rhizophora* | *Rhizophora racemosa* | nm | nm |
| Sapotaceae | *Manilkara* | *Manilkara huberi* | ms | mi |
| Sapotaceae | *Manilkara* | *Manilkara zapota* | ms | mi |
| Simaroubaceae | *Simarouba* | *Simarouba amara* | nm | nm |
| Solanaceae | *Solanum* | *Solanum lycocarpum* | ms | mi |
| Solanaceae | *Solanum* | *Solanum crinitum* | ms | mi |
| Velloziaceae | *Vellozia* | *Vellozia squamata* | nm | nm |
| Vochysiaceae | *Qualea* | *Qualea grandiflora* | nm | nm |
| Vochysiaceae | *Vochysia* | *Vochysia ferruginea* | nm | nm |

**Table S2 Neotropical plant species and the source included in the meta-analysis.**

| **Species** | **Reference source** |
| --- | --- |
| *Acrocomia aculeata* | Oliveira et al., 2012. Genetic diversity in populations of *Acrocomia aculeata* (Arecaceae) in the northern region of Minas Gerais, Brazil. Genetic Molecular Research 11: 531-538 |
| *Alcantarea geniculata* | Barbará et al., 2008. Within-population spatial genetic structure in four naturally fragmented species of a neotropical inselberg radiation, *Alcantarea imperialis*, *A. geniculata*, *A. glaziouana* and *A. regina* (Bromeliaceae). Heredity 101: 285-296 |
| *Alcantarea glaziouana* | Barbará et al., 2008. Within-population spatial genetic structure in four naturally fragmented species of a neotropical inselberg radiation, *Alcantarea imperialis*, *A. geniculata*, *A. glaziouana* and *A. regina* (Bromeliaceae). Heredity 101: 285-296 |
| *Alcantarea imperialis* | Barbará et al., 2008. Within-population spatial genetic structure in four naturally fragmented species of a neotropical inselberg radiation, *Alcantarea imperialis*, *A. geniculata*, *A. glaziouana* and *A. regina* (Bromeliaceae). Heredity 101: 285-296 |
| *Alcantarea regina* | Barbará et al., 2008. Within-population spatial genetic structure in four naturally fragmented species of a neotropical inselberg radiation, *Alcantarea imperialis*, *A. geniculata*, *A. glaziouana* and *A. regina* (Bromeliaceae). Heredity 101: 285-296 |
| *Anacardium humile* | Soares et al., 2013. Transferability and characterization of microssatellite loci in *Anacardium humile* A. St. Hil. (Anacardiaceae). Genetics and Molecular Research 12: 3146-3149 |
| *Annona cherimola* | van Zonneveld et al., 2012. Mapping genetic diversity of Cherimoya (*Annona cherimola* Mill.): Application of spatial analysis for conservation and use of plant genetic resources. PLoS ONE 1, 7: e29845 |
| *Annona coriacea* | Ribeiro et al., 2014. Transferability and characterization of nuclear microsatellite markers in populations of *Annona coriacea* (Annonaceae), a tree from the Brazilian cerrado. Brazilian Journal of Botany 37: 353-536 |
| *Annona crassiflora* | Ribeiro et al., 2016. Climatic drivers of leaf traits and genetic divergence in the tree *Annona crassiflora*: a broad spatial survey in the Brazilian savannas. Global Change Biology 22: 3789-3803 |
| *Annona crassiflora* | Collevatti et al., 2014. Contrasting spatial genetic structure in *Annona crassiflora* populations from fragmented and pristine savannas. Plant and Systematics Evolution 300: 1719-1727 |
| *Attalea phalerata* | Choo et al., 2012. Consequences of frugivore-mediated seed dispersal for the spatial and genetic structures of a neotropical Palm. Molecular Ecology 21: 1019-1031 |
| *Avicennia bicolor* | Nettel et al., 2008. Genetic diversity enhanced by ancient introgression and secondary contact in East Pacific black mangroves. Molecular Ecology 17: 2680-2690 |
| *Avicennia germinans* | Mori et al., 2015. Multiple-geographic-scale genetic structure of two mangrove tree species: The roles of mating system, hybridization, limited dispersal and extrinsic factors. PLoS ONE, doi.org/10.1371/journal.pone.0118710 |
| *Avicennia schaueriana* | Mori et al., 2015. Multiple-geographic-scale genetic structure of two mangrove tree species: The roles of mating system, hybridization, limited dispersal and extrinsic factors. PLoS ONE, doi.org/10.1371/journal.pone.0118710 |
| *Bactris gasipaes* | Rodrigues et al., 2004. Novel microsatellite markers for *Bactris gasipaes* (Palmae). Molecular Ecology Notes 4: 575-576 |
| *Bagassa guianensis* | Arruda et al., 2015. Mating system and genetic diversity of progenies before and after logging: a case study of *Bagassa guianensis* (Moraceae), a low-density dioecious tree of the Amazonian forest. Tree Genetics & Genomes 11: 3, doi:10.1007/s11295-015-0837-2 |
| *Butia eriospatha* | Nazareno and Reis, 2012. Linking phenology to mating system: exploring the reproductive biology of the threatened palm species *Butia eriospatha*. Journal of Heredity 103: 842-852 |
| *Carapa guianensis* | Cloutier et al., 2007. Low inbreeding and high pollen dispersal distances in populations of two amazonian forest tree species. Biotropica 39: 406-415 |
| *Carapa guianensis* | Dayanandan et al., 1999. Population structure delineated with microsatellite markers in fragmented populations of a tropical tree, *Carapa guianensis* (Meliaceae). Molceular Ecology 8: 1585-1592 |
| *Caryocar brasiliense* | Collevatti et al., 2001. Population genetic structure of the endangered tropical tree species *Caryocar brasiliense*, based on variability at microsatellite loci. Molecular Ecology 10: 349-356 |
| *Caryocar brasiliense* | Moura FN. 2011. Thesis: Caracterização de frutos e progênies de pequizeiro (*Caryocar brasiliense* camb.) do Cerrado |
| *Caryocar microcarpum* | Collevatti et al., 2009. Contrasting patterns of genetic structure in *Caryocar* (Caryocaraceae) congeners from flooded and upland Amazonian forests. Botanical Journal of the Linnean Society 98: 278-290 |
| *Caryocar villosum* | Collevatti et al., 2009. Contrasting patterns of genetic structure in *Caryocar* (Caryocaraceae) congeners from flooded and upland Amazonian forests. Botanical Journal of the Linnean Society 98: 278-290 |
| *Cattleya liliputana* | Leles et al., 2015. Genetic structure is associated with phenotypic divergence in floral traits and reproductive investment in a high-altitude orchid from the iron quadrangle, Southeastern Brazil. PLoS ONE, DOI:10.1371/journal.pone.0120645 |
| *Cedrela fissilis* | Mangaravite R, 2012. Thesis: Estrutura e diversidade genética no complexo *Cedrela fissilis* (Meliaceae) estimadas com marcadores microssatélites |
| *Cedrela odorata* | Cavers et al., 2003. A combination of molecular markers identifies evolutionarily significant units in *Cedrela odorata* L. (Meliaceae) in Costa Rica. Conservation Genetics 4: 571-580 |
| *Ceroxylon echinulatum* | Trénel et al., 2008. Landscape genetics, historical isolation and cross-Andean gene flow in the wax Palm, *Ceroxylon echinulatum* (Arecaceae). Molecular Ecology 17: 528-3540 |
| *Chamaedorea ernesti-augusti* | Cibrián-Jaramillo et al., 2009. Population genetics of the understory fishtail Palm *Chamaedorea ernesti-augusti* in Belize: high genetic connectivity with local differentiation. BMC Genetics 10: 65 |
| *Copaifera langsdorffii* | Carvalho and Oliveira, 2004. Genetic structure of *Copaifera langsdorffi* Desf. natural populations. Cerne 2: 137-153 |
| *Copaifera langsdorffii* | Tarazi R, 2009. Thesis: Diversidade genética, estrutura genética espacial, sistema de reprodução e fluxo gênico em uma população de *Copaifera langsdorffii* Desf. no cerrado |
| *Copaifera langsdorffii* | Lmor et al., 2014. Genetic structure and diversity of *Copaifera langsdorffii* desf. in Cerrado fragments of the São Paulo State, Brazil. Revista Árvore 38: 667-675 |
| *Curatella americana* | Canuto JZ, 2011. Thesis: Filogeografia de *Curatella americana* L. (Dilleniaceae): uma espécie arbórea das savanas da Amazônia e Brasil Central |
| *Dalbergia nigra* | Resende et al., 2011. Diversity and genetic connectivity among populations of a threatened tree (*Dalbergia nigra*) in a recently fragmented landscape of the Brazilian Atlantic Forest. Genetica 139: 1159-116 |
| *Dalbergia nigra* | Leite et al., 2014. Fine-scale genetic structure of the threatened rosewood *Dalbergia nigra* from the Atlantic Forest: comparing saplings versus adults and small fragment versus continuous forest. Tree Genetics & Genomes 10: 307-316 |
| *Dicorynia guianensis* | Latouche-Hallé et al., 2004. Long-distance pollen flow and tolerance to selfing in a neotropical tree species. Molecular Ecology 13: 1055-1064 |
| *Dimorphandra mollis* | Souza et al., 2012. Development of microsatellite markers for *Dimorphandra mollis* (Leguminosae), a widespread tree from the Brazilian Cerrado. American Journal of Botany: e102–e104 |
| *Dimorphandra mollis* | Souza et al., 2016. A large historical refugium explains spatial patterns of genetic diversity in a Neotropical savanna tree species. Annals of Botany 119: 239-252 |
| *Dimorphandra wilsonii* | Vinson et al., 2015. Population genetics of the naturally rare tree *Dimorphandra wilsonii* (Caesalpinioideae) of the Brazilian Cerrado. Tree Genetics & Genomes 11: 46 |
| *Dinizia excelsa* | Dick et al., 2003. Pollen dispersal of tropical trees (*Dinizia excelsa*: Fabaceae) by native insects and African honeybees in pristine and fragmented Amazonian rainforest. Molecular Ecology 12:753-764 |
| *Diospyros hispida* | Ibanes B, 2012. Thesis: Ecologia e diversidade genética de Diospyros hispida Alph. D.C. em duas áreas de cerrado no Estado de São Paulo. |
| *Dipteryx alata* | Tarazi et al., 2010. High levels of genetic differentiation and selfing in the Brazilian cerrado fruit tree *Dipteryx alata* Vog. (Fabaceae). Genetic Molecular Biology 33: 78-85 |
| *Dipteryx alata* | Diniz-Filho et al., 2012. Planning for optimal conservation of geographical genetic variability within species. Conservation Genetics 13: 1085-1093 |
| *Dipteryx panamensis* | Hanson et al., 2008. Pollen dispersal and genetic structure of the tropical tree *Dipteryx panamensis* in a fragmented Costa Rican landscape. Molecular Ecology 17: 2060-2073 |
| *Encholirium horridum* | Hmelejevski et al., 2015. Patterns of gene flow in *Encholirium horridum* L.B.Sm., a monocarpic species of bromeliaceae from Brazil. Journal of Heredity 106: 93-101 |
| *Epidendrum cinnabarinum* | Pinheiro et al., 2014. Rock outcrop orchids reveal the genetic connectivity and diversity of inselbergs of northeastern Brazil. BMC Evolutionary Biology 14: 49 |
| *Epidendrum fulgens* | Pinheiro et al., 2010. Hybridization and introgression across different ploidy levels in the Neotropical orchids *Epidendrum fulgens* and *E. puniceoluteum* (Orchidaceae). Molecular Ecology 19: 3981-3994 |
| *Epidendrum puniceoluteum* | Pinheiro et al., 2010. Hybridization and introgression across different ploidy levels in the Neotropical orchids *Epidendrum fulgens* and *E. puniceoluteum* (Orchidaceae). Molecular Ecology 19: 3981-3994 |
| *Epidendrum secundum* | Pinheiro et al., 2014. Rock outcrop orchids reveal the genetic connectivity and diversity of inselbergs of northeastern Brazil. BMC Evolutionary Biology 14: 49 |
| *Eugenia brasiliensis* | Ramos et al., 2014. Genetic diversity assessment for *Eugenia uniflora* L., *E. pyriformis* Cambess., *E. brasiliensis* Lam. and *E. francavilleana* O. Berg neotropical tree species (Myrtaceae) with heterologous SSR markers. Genetic Resources and Crop Evolution 61: 267-272 |
| *Eugenia dysentrica* | Zucchi et al., 2003. Genetic structure and gene flow in *Eugenia dysenterica* DC in the Brazilian Cerrado utilizing SSR markers. Genetics and Molecular Biology 26: 449-457 |
| *Eugenia dysentrica* | Barbosa et al., 2015. Range-wide genetic differentiation of *Eugenia dysenterica* (Myrtaceae) populations in Brazilian Cerrado. Biochemical Systematics and Ecology 59: 288e296 |
| *Eugenia francavilleana* | Ramos et al., 2014. Genetic diversity assessment for *Eugenia uniflora* L., *E. pyriformis* Cambess., *E. brasiliensis* Lam. and *E. francavilleana* O. Berg neotropical tree species (Myrtaceae) with heterologous SSR markers. Genetic Resources and Crop Evolution 61: 267-272 |
| *Eugenia pyriformis* | Ramos et al., 2014. Genetic diversity assessment for *Eugenia uniflora* L., *E. pyriformis* Cambess., *E. brasiliensis* Lam. and *E. francavilleana* O. Berg neotropical tree species (Myrtaceae) with heterologous SSR markers. Genetic Resources and Crop Evolution 61: 267-272 |
| *Eugenia uniflora* | Ramos et al., 2014. Genetic diversity assessment for *Eugenia uniflora* L., *E. pyriformis* Cambess., *E. brasiliensis* Lam. and *E. francavilleana* O. Berg neotropical tree species (Myrtaceae) with heterologous SSR markers. Genetic Resources and Crop Evolution 61: 267-272 |
| *Euterpe edulis* | Gaiotto et al., 2003. Genetic structure, mating system, and long-distance gene flow in heart of palm (*Euterpe edulis* mart.). Journal of Heredity 94: 399-406 |
| *Euterpe edulis* | Conte et al., 2006. Effects of management on the genetic structure of *Euterpe edulis* Mart. populations based on microsatellites. Scientia Forestalis 72: 81-88 |
| *Euterpe edulis* | Vieira et al., 2010. Spatial pattern and fine-scale genetic structure indicating recent colonization of the palm *Euterpe edulis* in a Brazilian Atlantic forest fragment. Biochemical Genetics 48: 96-10 |
| *Ficus citrifolia* | Heer et al., 2015. Spatial scales of genetic structure in free-standing and strangler figs (*Ficus*, Moraceae) inhabiting Neotropical forests. PLoS ONE, DOI:10.1371/journal.pone.0133581 |
| *Ficus insipida* | Heer et al., 2015. Spatial scales of genetic structure in free-standing and strangler figs (*Ficus*, Moraceae) inhabiting Neotropical forests. PLoS ONE, DOI:10.1371/journal.pone.0133581 |
| *Ficus obtusifolia* | Heer et al., 2015. Spatial scales of genetic structure in free-standing and strangler figs (*Ficus*, Moraceae) inhabiting Neotropical forests. PLoS ONE, DOI:10.1371/journal.pone.0133581 |
| *Ficus yoponensis* | Heer et al., 2015. Spatial scales of genetic structure in free-standing and strangler figs (*Ficus*, Moraceae) inhabiting Neotropical forests. PLoS ONE, DOI:10.1371/journal.pone.0133581 |
| *Gomortega keule* | Tonya et al., 2010. Fragmented but not isolated: Contribution of single trees, small patches and long-distance pollen flow to genetic connectivity for *Gomortega keule*, an endangered Chilean tree. Biological Conservation 143: 2583-2590 |
| *Handroanthus chrysotrichus* | Collevatti et al., 2014. High genetic diversity and contrasting fine-scale spatial genetic structure in four seasonally dry tropical forest tree species. Plant Systematics and Evolution 300: 1671-1681 |
| *Handroanthus impetiginosus* | Collevatti et al., 2014. High genetic diversity and contrasting fine-scale spatial genetic structure in four seasonally dry tropical forest tree species. Plant Systematics and Evolution 300: 1671-1681 |
| *Handroanthus serratifolius* | Collevatti et al., 2014. High genetic diversity and contrasting fine-scale spatial genetic structure in four seasonally dry tropical forest tree species. Plant Systematics and Evolution 300: 1671-1681 |
| *Himatanthus drasticus* | Baldauf et al., 2014. Genetic diversity, spatial genetic structure and realised seed and pollen dispersal of *Himatanthus drasticus* (Apocynaceae) in the Brazilian savanna. Conservation Genetics 15: 1073-1083 |
| *Himatanthus drasticus* | Baldauf et al., 2011. Characterization of microsatellite loci in *Himatanthus drasticus* (Apocynaceae), a medicinal plant from the Brazilian savanna. American Journal of Botany: e244–e246 |
| *Hymenaea courbaril* | Lacerda et al., 2008. Effects of reduced impact logging on genetic diversity and spatial genetic structure of a *Hymenaea courbaril* population in the Brazilian Amazon Forest. Forest Ecology and Management 255: 1034-1043 |
| *Hymenaea stigonocarpa* | Moraes and Sebben, 2011. Pollen dispersal between isolated trees in the Brazilian savannah: a case study of the Neotropical tree *Hymenaea stigonocarpa*. Biotropica 43: 192-199 |
| *Jacaranda copaia* | Jones and Hubbell, 2006. Demographic spatial genetic structure of the Neotropical tree, *Jacaranda copaia*. Molecular Ecology 15: 3205-3217 |
| *Jacaranda copaia* | Leclerc et al., 2015. Life after disturbance (I): changes in the spatial genetic structure of *Jacaranda copaia* (Aubl.) D. Don (Bignonianceae) after logging in an intensively studied plot in French Guiana. Annals of Forest Science 72: 509-516 |
| *Luehea seemannii* | Jones et al., 2004. Eight microsatellite markers for the neotropical tree *Luehea seemannii* (Tiliaceae). Molecular Ecology 4: 5-8 |
| *Manilkara huberi* | Azevedo et al., 2007. Genetic structure and mating system of *Manilkara huberi* (Ducke) A. Chev., a heavily logged amazonian timber species. Journal of Heredity 98: 646-654 |
| *Manilkara zapota* | Thompson et al., 2015. Genetic variation and structure in the neotropical tree, *Manilkara zapota* (L) P. Royen (Sapotaceae) used by the ancient Maya. Tree Genetics & Genomes 11: 40 |
| *Mauritia flexuosa* | Menezes et al., 2012. Development and characterization of DNA microsatellite primers for buriti (*Mauritia flexuosa* L.f.). Genetics and Molecular Research 11: 4058-4062 |
| *Miconia affinis* | Castilla et al., 2016. Elevation, not deforestation, promotes genetic differentiation in a pioneer tropical tree. PLoS ONE 11(6): e0156694. doi:10.1371/journal.pone.0156694 |
| *Myracrodruon urundeuva* | Moraes et al., 2005. Diversidade e estrutura genética espacial em duas populações de *Myracrodruon urundeuva* Fr. All. sob diferentes condições antrópicas. Revista Árvore 29: 281-289 |
| *Oenocarpus bataua* | Browne et al., 2015. Short-term genetic consequences of habitat loss and fragmentation for the Neotropical palm *Oenocarpus bataua*. Heredity 115: 389-395 |
| *Orthophytum ophiuroides* | Aoki-Gonçalves et al., 2014. Microsatellite loci for *Orthophytum ophiuroides* (Bromelioideae, Bromeliaceae) species adapted to Neotropical rock outcrops. Applications in Plant Sciences 2 (3): 1300073 |
| *Parkia panurensis* | Bialozyt et al., 2014. Primate seed dispersal leaves spatial genetic imprint throughout subsequent life stages of the Neotropical tree *Parkia panurensis*. Trees 28: 1569-1575 |
| *Pilosocereus aurisetus* | Bonatelli et al., 2014. Interglacial microrefugia and diversification of a cactus species complex: phylogeography and paleodistributional reconstructions for *Pilosocereus aurisetus* and allies. Molecular Ecology 20: 3185-3201 |
| *Pilosocereus machirisii* | Bonatelli et al., 2015. Using next generation RAD sequencing to isolate multispecies microsatellites for *Pilosocereus* (Cactaceae). PLoS ONE 10(11): e0142602.doi:10.1371/journal.pone.0142602 |
| *Plathymenia reticulata* | Cruz et al., 2012. Isolation and characterization of microsatellite markers for *Plathymenia reticulata* (Fabaceae). American Journal of Botany: e210–e212 |
| *Platypodium elegans* | Hufford et al., 2003. Viability selection at three early life stages of the tropical tree, *Platypodium elegans* (Fabaceae, Papilionoideae). Evolution 57: 518-526 |
| *Podocarpus sellowii* | Dantas et al., 2015. Low genetic diversity and high differentiation among relict populations of the Neotropical gymnosperm *Podocarpus sellowii* (Klotz.) in the Atlantic Forest. Genetica 143: 21-30 |
| *Polaskia chichipe* | Otero-Arnaiz et al., 2005. Genetic variation and evolution of *Polaskia chichipe* (Cactaceae) under domestication in the Tehuacán Valley, central Mexico. Molecular Ecology 14: 1603-1611 |
| *Pouteria reticulata* | Schroeder et al., 2014. Fine scale spatial genetic structure in *Pouteria reticulate* (Engl.) Eyma (Sapotaceae), a dioecious, vertebrate dispersed tropical rain forest tree species. Global Ecology and Conservation 1: 43-49 |
| *Qualea grandiflora* | Antiqueira and Kageyama, 2014. Genetic diversity of four populations of *Qualea grandiflora* Mart. in fragments of the Brazilian Cerrado. Genetica 142 :11-21 |
| *Qualea grandiflora* | Antiqueira and Kageyama, 2015. Reproductive system and pollen flow in progenies of *Qualea grandiflora* Mart., a typical species of the Brazilian Cerrado. Revista Árvore 39: 337-344 |
| *Rhizophora harrisonii* | Cerón-Souza et al., 2010. Hybridization and introgression in new world red Mangroves, *Rhizophora* (Rhizophoraceae). American Journal of Botany 97: 945-957 |
| *Rhizophora mangle* | Bruschi et al., 2014. Genetic and morphological variation of *Rhizophora mangle* (red mangrove) along the northern Pacific coast of Nicaragua. Nordic Journal of Botany 32: 320-329 |
| *Rhizophora racemosa* | Bruschi et al., 2014. Genetic and morphological variation of *Rhizophora mangle* (red mangrove) along the northern Pacific coast of Nicaragua. Nordic Journal of Botany 32: 320-329 |
| *Sextonia rubra* | Cloutier et al., 2007. Low inbreeding and high pollen dispersal distances in populations of two amazonian forest tree species. Biotropica 39: 406-415 |
| *Simarouba amara* | Hardesty et al., 2010. Geographic influence on genetic structure in the widespread neotropical tree *Simarouba amara* (Simaroubaceae). Tropical Plant Biology 3: 28-39 |
| *Solanum crinitum* | Moura et al., 2009. Diversidade e estrutura genética espacial em populações fragmentadas de *Solanum* spp. do Cerrado, estimadas por meio de locos microssatélites. Scientia Agricola 82: 143-15 |
| *Solanum lycocarpum* | Martins et al., 2006. Mating system and fine-scale spatial genetic structure of *Solanum lycocarpum* St.Hil. (Solanaceae) in the Brazilian Cerrado. Conservation Genetics 7: 957-969 |
| *Stenocereus stellatus* | Cruse-Sanders et al., 2013. Managing diversity: Domestication and gene flow in *Stenocereus stellatus* Riccob. (Cactaceae) in Mexico. Ecology and Evolution 3: 1340-1355 |
| *Swietenia humilis* | Rosas et al., 2011. Effects of habitat fragmentation on pollen flow and genetic diversity of the endangered tropical tree *Swietenia humilis* (Meliaceae). Biological Conservation 144: 3082-3088 |
| *Swietenia macrophylla* | Novick et al., 2003. Genetic structure of Mesoamerican populations of Big-leaf mahogany (*Swietenia macrophylla*) inferred from microsatellite analysis. Molecular Ecology 12: 2885-2893 |
| *Swietenia macrophylla* | Alcalá et al., 2015. Genetic structure and genetic diversity of *Swietenia macrophylla* in areas subjected to selective logging in Quintana Roo, Mexico. Botanical Sciences 93: 819-828 |
| *Swietenia macrophylla* | Alcalá et al., 2014. Genetic structure and genetic diversity of *Swietenia macrophylla* (Meliaceae): implications for sustainable forest management in Mexico. Journal of Tropical Forest Science 26: 142-152 |
| *Symphonia globulifera* | Aldrich et al., 1998. Microsatellite analysis of demographic genetic structure in fragmented populations of the tropical tree *Symphonia globulifera*. Molecular Ecology 7: 933-944 |
| *Tabebuia aurea* | Collevatti et al., 2014. Recovering species demographic history from multi-model inference: the case of a Neotropical savanna tree species. BMC Evolutionary Biology 14: 213 |
| *Tabebuia aurea* | Silva MC, 2010. Thesis: Diversidade genética, sistema de reprodução, estrutura genética espacial e fluxo gênico em *Tabebuia aurea* (Silva Manso) Benth. & Hook. f. ex S. Moore no Cerrado |
| *Handroanthus ochraceus* | Moreira et al., 2009. Fragmentation and spatial genetic structure in *Tabebuia ochracea* (Bignoniaceae) a seasonally dry Neotropical tree. Forest Ecology and Management 258: 2690-2695 |
| *Tabebuia roseoalba* | Feres et al., 2012. Mating system parameters at hierarchical levels of fruits, individuals and populations in the Brazilian insect-pollinated tropical tree, *Tabebuia roseo-alba* (Bignoniaceae) |
| *Tabebuia roseoalba* | Collevatti et al., 2014. High genetic diversity and contrasting fine-scale spatial genetic structure in four seasonally dry tropical forest tree species. Plant Systematics and Evolution 300: 1671-1681 |
| *Theobroma grandiflorum* | Alves et al., 2007. High levels of genetic divergence and inbreeding in populations of cupuassu (*Theobroma grandiflorum*). Tree Genet. Genomes 3: 289-298 |
| *Triplaris cumingiana* | Wie and Dick, 2014. Polymorphic microsatellite markers for a wind-dispersed tropical tree species, *Triplaris cumingiana* (Polygonaceae). Applications in Plant Sciences 2: 1400051 |
| *Uebelmannia pectinifera* | Moraes et al., 2014. Polymorphic microsatellite markers for the rare and endangered cactus *Uebelmannia pectinifera* (Cactaceae) and its congeneric species. Genetics and Molecular Research 13: 10359-10366 |
| *Vellozia squamata* | Silva MDB, 2013. Thesis: Diversidade e estrutura genética populacional de *Vellozia squamata* Pohl sob diferentes frequências de fogo no Cerrado. |
| *Virola koschnyi* | Riba-Hernández et al., 2014. Population and genetic structure of two dioecious timber species *Virola surinamensis* and *Virola koschnyi* (Myristicaceae) in southwestern Costa Rica. Forest Ecology and Management 323: 168-176 |
| *Virola surinamensis* | Riba-Hernández et al., 2014. Population and genetic structure of two dioecious timber species *Virola surinamensis* and *Virola koschnyi* (Myristicaceae) in southwestern Costa Rica. Forest Ecology and Management 323: 168-176 |
| *Vochysia ferruginea* | Davies et al., 2015. Pollen flow in fragmented landscapes maintains genetic diversity following stand-replacing disturbance in a Neotropical pioneer tree, *Vochysia ferruginea* Mart. Heredity 115: 125-129 |
| *Vouacapoua americana* | Dutech et al., 2004. Gene flow, historical population dynamics and genetic diversity within French Guianan populations of a rainforest tree species, *Vouacapoua americana*. Heredity 92: 69-77 |
| *Vriesea minarum* | Lavor et al., 2014. Population genetics of the endemic and endangered *Vriesea minarum* (Bromeliaceae) in the iron quadrangle, Espinhaço Range, Brazil. American Journal of Botany 101: 1167-1175 |

**Table S3**. **Phylogenetic non-independent vs. phylogenetic-independent random-effect meta-analysis for mammal-dispersed species dataset based on microsatellite markers, comparing (a) megafauna seed dispersal syndromes and no megafauna syndromes (ms vesus nm), and (b)** **megafauna dependent (md), megafauna independent (mi) and no megafauna syndromes (nm) (md vs mi vs nm).** The parameters are *F_ST_*, genetic differentiation; *He*, genetic diversity; *AR*, allelic richness. k, number of species compared; δ, mean pooled effect size; δ^2^ , variance of effect size; 95% CI confidence interval of effect size; AICc is the Akaike information criterion corrected for sample size and number os parameters. Z –test for non-zero effect sizes. Z values are significant, p < 0.001. In bold are the lowest AIC value for ordinary and phylogenetic meta-analyses comparisons.

|  |  |  |  | **Phylogenetic non-independent** | | | |  |  | **Phylogenetic-independent** | | | |
| --- | --- | --- | --- | --- | --- | --- | --- | --- | --- | --- | --- | --- | --- |
| **Parameter** | **k** | **δ** | **δ^2^** | **95% CI** | **Z** | **p** | **AICc** | **δ** | **δ^2^** | **95% CI** | **Z** | **p** | **AICc** |
| **(a) *ms v nm*** |  |  |  |  |  |  |  |  |  |  |  |  |  |
| ***F_ST_*** | 18 | 0.138 | 0.0012 | 0.070 - 0.207 | 15.57 | 0.0001 | **49.45** | 0.138 | 0.0012 | 0.069 - 0.207 | 15.44 | 0.0001 | 52.36 |
| ***He*** | 41 | 0.651 | 0.0006 | 0.602 - 0.700 | 671.91 | 0.0001 | **117.69** | 0.652 | 0.0006 | 0.602 - 0.702 | 661.10 | 0.0001 | 119.98 |
| ***AR*** | 21 | 5.539 | 0.4407 | 4.238 - 6.841 | 69.63 | 0.0001 | **59.62** | 5.539 | 0.4407 | 4.238 - 6.840 | 69.62 | 0.0001 | 62.08 |
| **(b) *md v mi v nm*** |  |  |  |  |  |  |  |  |  |  |  |  |  |
| ***F_ST_*** | 18 | 0.138 | 0.0013 | 0.067 - 0.209 | 14.60 | 0.0001 | **48.14** | 0.138 | 0.0013 | 0.067 - 0.209 | 14.48 | 0.0001 | 51.05 |
| ***He*** | 41 | 0.651 | 0.0006 | 0.603 - 0.699 | 696.95 | 0.0001 | **116.21** | 0.652 | 0.0006 | 0.603 - 0.701 | 685.46 | 0.0001 | 118.43 |
| ***AR*** | 21 | 5.541 | 0.4570 | 4.216 - 6.866 | 67.18 | 0.0001 | **58.67** | 5.539 | 0.4407 | 4.215 - 6.865 | 60.17 | 0.0001 | 61.13 |

**Table S4. Phylogenetic non-independent vs. phylogenetic-independent random-effects meta-analysis comparison for zoochoric species dataset, based on microsatellites markers comparing (a) megafauna seed dispersal syndromes and no megafauna syndromes (ms vesus nm), and (b)** **megafauna dependent (md), megafauna independent (mi) and no megafauna syndromes (nm) (md vs mi vs nm).** The parameters are *F_ST_*, genetic differentiation; *He*, genetic diversity; *AR*, allelic richness. k, number of species compared; δ, mean pooled effect size; δ^2^ , variance of effect size; 95% CI confidence interval of effect size; AICc is the Akaike information criterion corrected for sample size and number os parameters. Z –test for non-zero effect sizes. Z values are significant, p < 0.001. In bold are the lowest AIC value for ordinary and phylogenetic meta-analyses comparisons.

|  |  |  |  | **Phylogenetic non-independent** | | | |  |  | **Phylogenetic independent** | | | | |
| --- | --- | --- | --- | --- | --- | --- | --- | --- | --- | --- | --- | --- | --- | --- |
| **Parameter** | **k** | **δ** | **δ^2^** | **95% CI** | **Z** | **p** | **AICc** | **δ** | **δ^2^** | **95% CI** | **Z** | **p** | **AICc** |  |
| **(a) *ms v nm*** |  |  |  |  |  |  |  |  |  |  |  |  |  |  |
| ***F_ST_*** | 27 | 0.210 | 0.0020 | 0.123 - 0.297 | 22.36 | 0.0001 | **75.52** | 0.210 | 0.0020 | 0.123 - 0.297 | 22.25 | 0.0001 | 78.06 |  |
| ***He*** | 51 | 0.641 | 0.0014 | 0.597 - 0.685 | 811.13 | 0.0001 | **146.48** | 0.641 | 0.0014 | 0.596 - 0.686 | 786.92 | 0.0001 | 148.85 |  |
| ***AR*** | 24 | 5.192 | 0.3529 | 4.028 - 6.357 | 76.41 | 0.0001 | **67.58** | 5.192 | 0.3530 | 4.027 - 6.356 | 76.35 | 0.0001 | 69.99 |  |
| **(b) *md v mi v nm*** |  |  |  |  |  |  |  |  |  |  |  |  |  |  |
| ***F_ST_*** | 27 | 0.210 | 0.0020 | 0.122 - 0.299 | 21.61 | 0.0001 | **73.99** | 0.210 | 0.0021 | 0.121 - 0.299 | 21.50 | 0.0001 | 76.53 |  |
| ***He*** | 51 | 0.641 | 0.0005 | 0.598 - 0.685 | 834.34 | 0.0001 | **145.00** | 0.641 | 0.0005 | 0.597 - 0.685 | 808.97 | 0.0001 | 147.41 |  |
| ***AR*** | 24 | 5.194 | 0.3650 | 4.010 - 6.379 | 73.92 | 0.0001 | **66.60** | 5.194 | 0.3652 | 4.009 - 6.378 | 73.86 | 0.0001 | 69.01 |  |

**Table S5. Phylogenetic non-independent vs. phylogenetic-independent random-effects meta-analysis comparison for all species dataset, based on microsatellites markers comparing (a) megafauna seed dispersal syndromes and no megafauna syndromes (ms vesus nm), and (b) megafauna dependent (md), megafauna independent (mi) and no megafauna syndromes (nm) (md vs mi vs nm).** The parameters are *F_ST_*, genetic differentiation; *He*, genetic diversity; *AR*, allelic richness. k, number of species compared; δ, mean pooled effect size; δ^2^ , variance of effect size; 95% CI confidence interval of effect size; AICc is the Akaike information criterion corrected for sample size and number os parameters. Z –test for non-zero effect sizes. Z values in bold are significant, p < 0.001. In bold are the lowest AIC value for ordinary and phylogenetic meta-analyses comparisons.

|  |  |  |  | **Phylogenetic non-independent** | | | |  |  | **Phylogenetic independent** | | | |
| --- | --- | --- | --- | --- | --- | --- | --- | --- | --- | --- | --- | --- | --- |
| **Parameter** | **k** | **δ** | **δ^2^** | **95% CI** | **Z** | **p** | **AICc** | **δ** | **δ^2^** | **95% CI** | **Z** | **p** | **AICc** |
| ***(a)ms v nm*** |  |  |  |  |  |  |  |  |  |  |  |  |  |
| ***F_ST_*** | 55 | 0.213 | 0.0009 | 0.154 - 0.273 | 49.65 | 0.0001 | **150.34** | 0.213 | 0.0020 | 0.153 - 0.273 | 48.08 | 0.0001 | 152.68 |
| ***He*** | 85 | 0.649 | 0.0004 | 0.611 - 0.687 | 1112.47 | 0.0001 | **238.24** | 0.647 | 0.0004 | 0.608 - 0.687 | 1040.24 | 0.0001 | 240.59 |
| ***AR*** | 56 | 5.414 | 0.1602 | 4.630 - 6.199 | 182.96 | 0.0001 | **157.61** | 5.412 | 0.1690 | 4.626 - 6.198 | 182.04 | 0.0001 | 159.89 |
| ***(b)md v mi v nm*** |  |  |  |  |  |  |  |  |  |  |  |  |  |
| ***F_ST_*** | 55 | 0.213 | 0.0009 | 0.153 - 0.273 | 48.60 | 0.0001 | **149.14** | 0.213 | 0.0010 | 0.152 - 0.274 | 47.07 | 0.0001 | 151.48 |
| ***He*** | 85 | 0.649 | 0.0004 | 0.611 - 0.687 | 1124.30 | 0.0001 | **236.73** | 0.647 | 0.0004 | 0.608 - 0.687 | 1050.88 | 0.0001 | 239.16 |
| ***AR*** | 56 | 5.414 | 0.1622 | 4.625 - 6.204 | 180.71 | 0.0001 | **156.71** | 5.412 | 0.1629 | 4.621 - 6.204 | 179.80 | 0.0001 | 158.98 |

**Table S6**. **Phylogenetic non-independent random-effects meta-analysis for each dataset based on microsatellite markers comparing (a) megafauna seed dispersal syndromes vs no megafauna syndromes (ms versus nm), (b) megafauna dependent (md) vs no megafauna syndromes (md versus nm), and (c) megafauna dependent (md) vs megafauna independent (md versus mi).** The parameters are *F_ST_*, genetic differentiation; *He*, genetic diversity; *AR*, allelic richness. k, number of species compared; MD, mean difference and 95% CI (confidence interval). Stat-Power refers to the statistical power of each of the treatment given the effect size and sample size; values in bold represents enough statistical power.

|  |  |  | **Mammals** |  |  |  | **Zoochoric** |  | **All species** | | |  |
| --- | --- | --- | --- | --- | --- | --- | --- | --- | --- | --- | --- | --- |
| **Parameter** | **k** | **MD** | **95% CI** | **Stat-Power** | **k** | **MD** | **95% CI** | **Stat-Power** | **k** | **MD** | **95% CI** | **Stat-Power** |
| 1. ***ms v nm*** |  |  |  |  |  |  |  |  |  |  |  |  |
| ***F_ST_*** | 10/8 | 0.0192 | -0.093 – 0.131 | 0.074 | 11/16 | -0.1030 | -0.244 – 0.037 | 0.138 | 12/43 | -0.0715 | -0.167 – 0.024 | 0.275 |
| ***He*** | 19/22 | -0.0443 | -0.135 – 0.046 | **0.948** | 19/32 | -0.0316 | -0.119 – 0.055 | **0.947** | 19/66 | -0.0445 | -0.125 – 0.036 | **0.999** |
| ***AR*** | 10/11 | -1.0682 | -3.859 – 1.723 | **1.000** | 9/15 | -0.7440 | -3.225 – 1.737 | **1.000** | 10/46 | -0.6098 | -2.881 – 1.661 | **1.000** |
| **(b) *md v nm*** |  |  |  |  |  |  |  |  |  |  |  |  |
| ***F_ST_*** | 7/8 | 0.020 | -0.120 – 0.170 | 0.050 | 7/16 | -0.100 | -0.260 – 0.070 | 0.084 | 8/43 | -0.070 | -0.200 – 0.060 | 0.131 |
| ***He*** | 11/22 | 0.020 | -0.090 – 0.130 | **0.581** | 11/32 | 0.030 | -0.070 – 0.140 | **0.663** | 11/66 | 0.020 | -0.080 – 0.120 | **0.875** |
| ***AR*** | 6/11 | -1.750 | -4.510 – 1.010 | **0.999** | 5/15 | -1.420 | -3.870 – 1.020 | **0.999** | 6/46 | -1.290 | -3.520 – 0.940 | **1.000** |
| **(c) *md v mi*** |  |  |  |  |  |  |  |  |  |  |  |  |
| ***F_ST_*** | 7/3 | -0.010 | -0.140 – 0.110 | 0.050 | 7/4 | -0.010 | -0.140 – 0.110 | 0.084 | 8/4 | -0.010 | -0.140 – 0.110 | 0.131 |
| ***He*** | 11/8 | 0.140 | 0.000 – 0.280 | **0.581** | 11/8 | 0.140 | 0.000 – 0.280 | **0.663** | 11/8 | 0.140 | 0.000 – 0.280 | **0.875** |
| ***AR*** | 6/4 | -1.630 | -6.470 – 3.210 | **0.999** | 5/4 | -1.630 | -6.470 – 3.210 | **0.999** | 6/4 | -1.630 | -6.470 – 3.210 | **1.000** |

**Table S7. Mean values of the posterior distribution of the GLMM for genetic parameters based on microsatellite markers for all dataset.** *F_ST_*, genetic differentiation; *He,* genetic diversity overall populations. Significant values are denoted in bold.

| Parameters | | Microsatellite | | | |
| --- | --- | --- | --- | --- | --- |
|  |  | ***F_ST_*** | | ***He*** | |
|  |  | **Mean** | **p-value** | **Mean** | **p-value** |
| Megafaunasyndrome | **Yes** | -0.023 | 0.721 | -0.027 | 0.579 |
|  | **No** | **-** | **-** | **-** | **-** |
| Pollination Mode | **Anemophily** | **-** | **-** | **-** | **-** |
|  | **Chiropterophily** | **-** | **-** | **-** | **-** |
|  | **Entomophily** | 0.089 | 0.285 | -0.052 | 0.411 |
|  | **Ornitophily** | -0.018 | 0.919 | -0.005 | 0.968 |
| Mating system | **Mixed** | **-** | **-** | **-** | **-** |
|  | **Outcrossing** | **-0.162** | **0.008** | **0.129** | **0.018** |
| Breeding system | **Dioecious** | **-** | **-** | **-** | **-** |
|  | **Monoecious** | 0.009 | 0.923 | 0.029 | 0.781 |
|  | **Hermaphrodite** | 0.030 | 0.785 | -0.011 | 0.884 |

**Table S8. Mean values of the posterior distribution of the GLMM for genetic parameters based on microsatellite markers for species dispersed by mammals**. *F_ST_*, genetic differentiation; *He,* genetic diversity overall populations. Significant values are denoted in bold.

| Parameter | | Microsatellites | | | |
| --- | --- | --- | --- | --- | --- |
|  |  | ***F_ST_*** | | ***He*** | |
|  |  | **Mean** | **p-value** | **Mean** | **p-value** |
| Megafauna  syndrome | **Yes** | -0.040 | 0.626 | -0.013 | 0.828 |
|  | **No** | **-** | **-** | **-** | **-** |
| Pollination Mode | **Anemophily** | **-** | **-** | **-** | **-** |
|  | **Chiropterophily** | **-** | **-** | **-** | **-** |
|  | **Entomophily** | 0.048 | 0.577 | **-0.146** | **0.033** |
|  | **Ornitophily** | -0.027 | 0.866 | 0.074 | 0.685 |
| Mating system | **Mixed** | **-** | **-** | **-** | **-** |
|  | **Outcrossing** | -0.125 | 0.161 | 0.056 | 0.443 |
| Breeding system | **Dioecious** | **-** | **-** | **-** | **-** |
|  | **Monoecious** | -0.025 | 0.816 | 0.023 | 0.911 |
|  | **Hermaphrodite** |  |  | -0.193 | 0.129 |

**Table S9. Mean values of the posterior distribution of the GLMM for genetic parameters based on microsatellite markers for species with zoochoric syndrome**. *F_ST_*, genetic differentiation; *He,* genetic diversity overall populations. Significant values are denoted in bold.

| Parameter | | Microsatellites | | | |
| --- | --- | --- | --- | --- | --- |
|  |  | ***F_ST_*** | | ***He*** | |
|  |  | **Mean** | **p-value** | **Mean** | **p-value** |
| Megafauna syndrome | **Yes** | -0.145 | 0.100 | 0.008 | 0.876 |
|  | **No** | **-** | **-** | **-** | **-** |
| Pollination Mode | **Anemophily** | **-** | **-** | **-** | **-** |
|  | **Chiropterophily** | **-** | **-** | **-** | **-** |
|  | **Entomophily** | 0.070 | 0.504 | **-0.151** | **0.025** |
|  | **Ornitophily** | -0.147 | 0.407 | -0.010 | 0.951 |
| Mating system | **Mixed** | **-** | **-** | **-** | **-** |
|  | **Outcrossing** | **-0.224** | **0.023** | 0.069 | 0.276 |
| Breeding system | **Dioecious** | **-** | **-** | **-** | **-** |
|  | **Monoecious** | 0.075 | 0.581 | 0.111 | 0.410 |
|  | **Hermaphrodite** | 0.137 | 0.288 | -0.092 | 0.207 |

**Table S10.** **Phylogenetic signal of megafauna seed dispersal syndromes and reproductive traits for Neotropical plants included in the meta-analyses using Abouheif’s proximity test of serial independence**. Values followed by *ns* are not significant, p > 0.05.

| **Trait** | **Observed Moran’s I** | **p-value** |
| --- | --- | --- |
| **Megafauna syndromes** | 0.044 | 0.001 |
| **Pollination mode** | 0.059 | 0.001 |
| **Breeding system** | 0.039 ^ns^ | 1.000 |
| **Mating system** | 0.025 ^ns^ | 1.000 |

**Table S11. Phylogenetic generalized least squares for genetic parameters based on microsatellite markers for all species dataset.** Significant values are denoted in bold. *F_ST_,* genetic differentiation; *Hes,* genetic diversity overall populations; SE, standard error.

|  | **Parameter** | ***F_ST_*** | | | ***He*** | | |
| --- | --- | --- | --- | --- | --- | --- | --- |
| **Trait** | **Variable** | **Coefficient±SE** | **t-value** | **p-value** | **Coefficient±SE** | **t-value** | **p-value** |
|  | **Intercept** | 0.225±0.201 | 1.123 | 0.267 | 0.396**±**0.149 | 2.664 | 0.010 |
| **Megafauna syndromes** | **Yes** | -0.030±0.086 | -0.353 | 0.726 | -0.015**±**0.056 | -0.268 | 0.790 |
|  | **No** | **-** | **-** | **-** | **-** | **-** | **-** |
| **Pollination mode** | **Anemophily** | - | - | - | **-** | **-** | **-** |
|  | **Chiropterophily** | - | - | - | **-** | **-** | **-** |
|  | **Entomophily** | 0.157±0.122 | 1.287 | 0.204 | -0.062**±**0.088 | -0.713 | 0.479 |
|  | **Ornitophily** | -0.061±0.155 | -0.394 | 0.695 | 0.028**±**0.119 | 0.235 | 0.815 |
| **Mating system** | **Mixed** | - | - | - |  |  |  |
|  | **Outcrossed** | **-0.188±0.065** | **-2.872** | **0.006** | **0.120±0.050** | **2.418** | **0.019** |
| **Breeding system** | **Dioecious** |  |  |  |  |  |  |
|  | **Monoecious** | 0.126±0.137 | 0.919 | 0.363 | 0.045**±**0.103 | 0.433 | 0.667 |
|  | **Hermaphrodite** | 0.068±0.114 | 0.596 | 0.554 | -0.036**±**0.094 | -0.379 | 0.707 |

**Table S12. Phylogenetic generalized least squares for genetic parameters based on microsatellite markers for zoochoric and mammal dispersed species.** Significant values are denoted in bold. *F_ST_,* genetic differentiation; *He,* genetic diversity overall populations; SE, standard error.

|  |  | **Zoochoric** | | | | | | **Mammals** | | |
| --- | --- | --- | --- | --- | --- | --- | --- | --- | --- | --- |
|  | **Parameter** | ***F_ST_*** | | | ***He*** | | | ***He*** | | |
| **Trait** | **Variable** | **Coefficient±SE** | **t-value** | **p-value** | **Coefficient±SE** | **t-value** | **p-value** | **Coefficient±SE** | **t-value** | **p-value** |
|  | **Intercept** | 0.497±0.309 | 1.609 | 0.120 | 0.537±0.145 | 3.715 | 0.001 | 0.977±0.201 | 4.865 | 0.001 |
| **Megafauna syndromes** | **Yes** | -0.125±0.108 | -1.149 | 0.261 | 0.024±0.053 | 0.448 | 0.657 | -0.056±0.074 | -0.748 | 0.462 |
|  | **No** | **-** | **-** | **-** | **-** | **-** | **-** | **-** | **-** | **-** |
| **Pollination mode** | **Anemophily** | - | - | - | - | - | - | - | - | - |
|  | **Chiropterophily** | **-** | **-** | **-** | **-** | **-** | **-** | **-** | **-** | **-** |
|  | **Entomophily** | -0.039±0.223 | -0.174 | 0.863 | -0.170±0.088 | -1.927 | 0.062 | -0.155±0.088 | -1.752 | 0.092 |
|  | **Ornitophily** | -0.161±0.189 | -0.850 | 0.403 | 0.023±0.107 | 0.216 | 0.830 | -0.012±0.162 | -0.072 | 0.943 |
| **Mating system** | **Mixed** | - | - | - |  |  |  |  |  |  |
|  | **Outcrossed** | **-0.250**±**0.108** | **-2.314** | **0.029** | 0.071±0.065 | 1.101 | 0.278 | 0.080±0.071 | 1.138 | 0.266 |
| **Breeding system** | **Dioecious** | **-** | **-** | **-** | - | - | - | - | - | - |
|  | **Monoecious** | 0.140±0.156 | 0.893 | 0.380 | 0.060±0.107 | 0.559 | 0.579 | -0.074±0.149 | -0.496 | 0.169 |
|  | **Hermaphrodite** | 0.130±0.136 | 0.956 | 0.348 | -0.049±0.082 | -0.602 | 0.551 | -0.182±0.128 | -1.419 | 0.625 |
