# Supplementary material for: Megafauna Seed Dispersal in the Neotropics: A Meta-Analysis Shows No Genetic Signal of Loss of Long-Distance Seed Dispersal
Source: Front Genet. 2019 Sep 5;10:788. doi: 10.3389/fgene.2019.00788 (PMC6739635; doi:10.3389/fgene.2019.00788)
Supplement: Supporting Information S2 — Figures S1 and S2 with information of meta-analysis results. [file Table_2.docx]

**Megafauna seed dispersal in the Neotropics: A meta-analysis shows no genetic signal of loss of long-distance seed dispersal**

Rosane G Collevatti*, Jacqueline S Lima, Liliana Ballesteros-Mejia

**Supporting Information S2 - Figures**


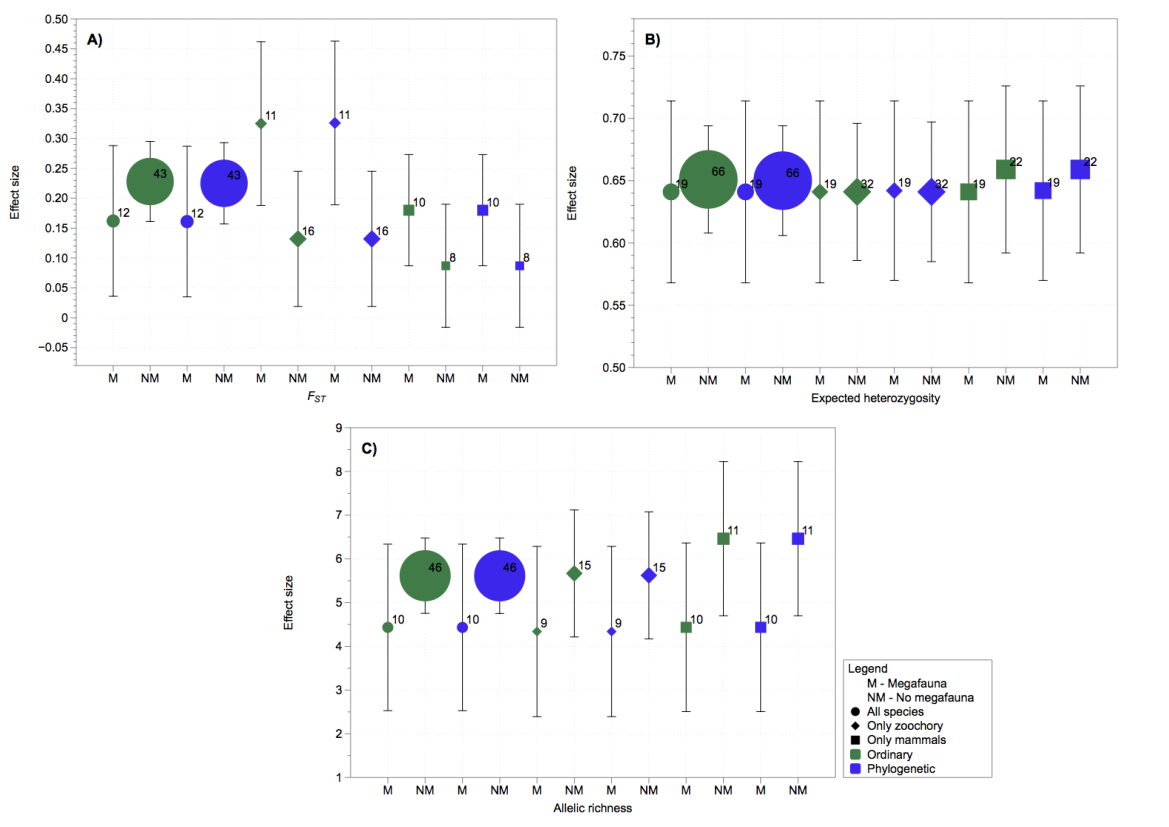


**Figure S1.** Comparison between ordinary and phylogenetic independent meta-analysis of the effect of megafauna seed dispersal syndromes on genetic parameters across the three data sets for plant species with megafauna and no megafauna syndromes. (A) Effect size in genetic differentiation (*F_ST_*). (B) Effect size in genetic diversity (expected heterozygosity, *He*). (C) Effect size in allelic richness (*AR*).


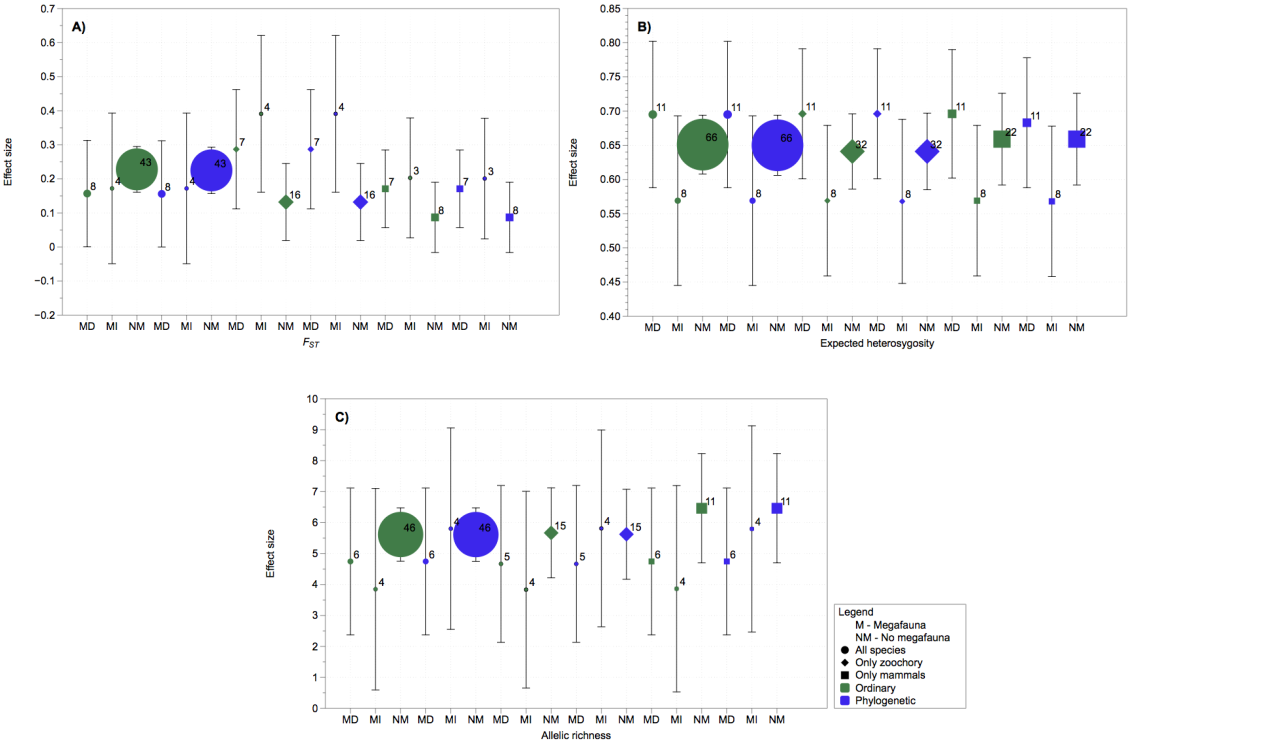


**Figure S2.** Comparison between ordinary and phylogenetic independent meta-analysis of the effect of megafauna seed dispersal syndromes on genetic parameters across the three data sets for plant species megafauna dependent, megafauna independent and with no megafauna syndrome. (A) Effect size in genetic differentiation (*F_ST_*). (B) Effect size in genetic diversity (expected heterozygosity, *He*). (C) Effect size in allelic richness (*AR*).
